# Food Authenticity Models for *Mytilus galloprovincialis* (Mediterranean Mussel): Exploratory Study

**DOI:** 10.3390/foods14244195

**Published:** 2025-12-06

**Authors:** Sandra Fernández Suárez, Javier Lorenzo Galbán, Sabela Fernandez-Sanchez, Maria Garcia-Marti, Gonzalo Astray

**Affiliations:** 1 Universidade de Vigo, Facultade de Ciencias, 32004 Ourense, Spain; sandra.fernandez.suarez@alumnado.uvigo.gal (S.F.S.); javier.lorenzo@alumnado.uvigo.gal (J.L.G.); 2 Universidade de Vigo, Departamento de Química Analítica e Alimentaria, Facultade de Ciencias, 32004 Ourense, Spain; sab.fernandez@uvigo.gal (S.F.-S.); maria.garcia.marti@uvigo.gal (M.G.-M.); 3 Universidade de Vigo, Departamento de Química Física, Facultade de Ciencias, 32004 Ourense, Spain

**Keywords:** mussel, stable isotope ratio, trace element, machine learning, random forest, support vector machine, artificial neural network

## Abstract

Geographical origin determination for seafood products is a fundamental aspect due to its implications for fraud prevention, ensuring food safety, and promoting resource sustainable management. In this research, different machine learning (ML) models based on random forests, support vector machines, and artificial neural networks were fed with trace element fingerprinting (TEF) and stable isotope ratio analysis (SIRA) to determine the origin of mussels that have been farmed in eight regions and ten locations around the world (areas of the European Atlantic coast, the Mediterranean Sea, and the Pacific coast of Chile). Fourteen trace elements in shells and carbon and nitrogen isotope ratios of mussel tissue were used singly, in combination, or reduced to develop the different approach models. All the selected models present high prediction accuracies for the independent variables (except for SIRA models), for their combination, or for their optimisation, highlighting the artificial neural network and random forest models that presented a 100% accuracy for all cases using a combination of variables selected based on a random forest model TEF to predict region and location, respectively. This fact confirms that ML models are suitable approximation techniques to determine the region and location of Mediterranean mussel origin, with key applications in food safety and global sustainability.

## 1. Introduction

In the food sector, ensuring the geographical origin of marine bivalves is essential to prevent fraud and ensure the quality of food demanded by consumers [1]. Bivalves play a significant role in human nutrition, so knowing the traceability of foods ensures sustainable management of these products, minimises food safety risks, and prevents fraudulent practices [2]. According to FAO (2024) data [3], total mussel production was almost 2 million tonnes in 2022, with Spain being the third largest generator [4]. In marine bivalve seafood, practices such as incorrect labelling compromise environmental, social, economic, and public health aspects [5]. Therefore, EU regulations require that food labelling for these products include the origin of the species, among other data [6].

Due to the need to identify the geographical origin of the food product, it has been necessary to develop analytical and prediction techniques that can obtain acceptable results while ensuring authenticity. One of the most widely used methods to track the traceability of seafood is the study of its stable isotope ratio analysis (SIRA), since the accumulation of certain elements in the body’s tissues provides information on the geographical distribution of different species [7]. Stable carbon (C) and nitrogen (N) isotopes (δ^13^C, δ^15^N) of mussels provide information on the habitat of these species [8]. On the other hand, trace element fingerprinting (TEF) in bivalve shells and soft tissues has been described as an accurate approach to identify the origin of *Mytilus edulis* [9]. However, although the use of biogeochemical signatures to research traceability has grown in recent years in these species, case studies applied in real contexts are still relatively scarce [10]. According to Varrà et al. (2023) [11], stable isotopic ratios and trace element analysis have gained popularity in recent years as one of the most promising techniques, given their potential to provide accurate information to find solutions for multiple authenticity issues, including the geographical origin of seafood [12]. However, the complexity of isotopic profiles provided valuable information for tracing the geographical origin; the results may be difficult to interpret due to insufficient variation between isotopic profiles [13]. On the other hand, multi-element signature scientific techniques have proven useful in determining the food origin of animal-derived foods; it is necessary to explore the combination of these techniques with other, more complex tools to improve the accuracy of the results [14]. Consequently, tools such as chemometrics and machine learning (ML) have gained prominence as methodologies to support these analytical techniques and achieve more robust outcomes [15]. According to Li et al. (2025) [16], machine learning methods provide advanced solutions for identifying, authenticating, and tracing the geographic origin of food, thanks to their ability to analyse complex datasets, learn meaningful patterns, and make accurate predictions for unknown samples [17]. These methodologies have been successfully applied for this purpose in different seafood, such as sea cucumber (*Apostichopus japonicus*) [18], Chinese oysters (*Crassostrea gigas*) [19], and mussels (*Mytilus edulis*, *Mytilus galloprovincialis*, and *Mytilus trossulus*) [20]. Furthermore, our research group has demonstrated that the use of machine learning methodologies such as random forest, support vector machine, and artificial neural network (RF, SVM and ANN, respectively) are tools that work adequately to classify the geographical origin of food products to ensure the traceability of these products and contribute to the safety of the food value chain.

The first machine learning models developed in the present research were random forest (RF) models. RF is a nonparametric model [21] that can be applied to both classification and regression problems [22,23]. Random forest uses a set of classification and regression trees (CARTS) for predictive purposes [24]. According to Z. Sun et al. (2024) [25], the random decision forest procedure was first proposed by Tim Kam Ho in 1995 [26], but it was in this century that the term was formally introduced by Breiman (2001) [27]. Random forest can achieve high accuracy across various types of datasets while maintaining computational efficiency [28]. In addition to all this, and according to Yates & Islam (2021), its performance has been further improved through parallelisation, enabling the construction of decision trees simultaneously on multi-core processor systems [28]. According to Pineda-Metz et al. (2023) [29], random forest models stand out for their performance and precision, their ability to work with large sets of independent variables, and their insensitivity to overfitting.

The second machine learning models developed were the support vector machine (SVM) models. Support vector machine is a supervised learning technique used for classification and regression purposes, as it optimises a separating hyperplane between classes using adjustable parameters, maximising accuracy and avoiding overtraining [30]. The support vector machine is a robust machine learning method that operates on the principle of minimising structural risk [31]. According to Wang et al. (2024) [32], support vector machines were introduced by Cortes & Vapnik (1995) [33]. SVM can be applied to so many tasks related to classification, since it works to classify the non-linear data with a linear decision surface using a kernel function [34]. Therefore, the SVM can be linear or non-linear, most of the time being linear, and its complexity depends on the quantity of characteristics used, as the training process involves finding a hyperplane that maximises the margin between the support vectors of the classes [35]. One principal strength of SVM models is their ability to deal with high-dimensional data, making them a good option for the analysis of complex data [36].

Finally, the artificial neural network (ANN) models were developed. The basis of these models is to simulate the human nervous system structure [37] to establish a relationship between input and output values [38]. They consist of neurons organised in different layers: an input layer, which receives data, and an output layer that generates a response, but they can also have other layers, such as hidden layers [39]. Typically, an ANN comprises an input layer, which receives signals from the dataset; one or more hidden layers disposed between the input and output layers; and an output layer that provides the final outcomes [39]. Although these types of models require a large amount of data, ANNs can extract relevant features and establish accurate models compared to other traditional machine learning methods [37].

To sum up, considering what was previously said about the three machine learning models regarding the advantages they offer to model data and their widespread use in different fields of science, in the present research, the development of prediction models based on these algorithms will be carried out to ensure the geographic origin of *Mytilus galloprovincialis* (Mediterranean mussel) based on their chemical signature obtained from their trace element fingerprinting (TEF) and stable isotopic composition analysis (SIRA). This research will also analyse the impact of using unbalanced classes and groups and show how this imbalance can affect the model’s results.

## 2. Materials and Methods

### 2.1. Experimental Data

The different samples of Mediterranean mussels (*Mytilus galloprovincialis*) from Chile, France, Italy, Portugal, and Spain were collected between September 2018 and September 2019, and the samples from Tunisia were collected in January 2018 [40]. Therefore, the total number of Mediterranean mussels covers six countries, corresponding to eight harvesting regions and ten location zones. The total number of samples obtained by del Rio-Lavín et al. (2022) [40] was distributed as follows: 100 samples were used for the determination of TEF in the shell, 179 samples were used for the determination of SIRA in the soft tissue; of all these samples, 64 had simultaneous data on SIRA and TEF.

Fourteen trace elements (^11^B, ^27^Al, ^47^Ti, ^51^V, ^52^Cr, ^55^Mn, ^59^Co, ^60^Ni, ^63^Cu, ^66^Zn, ^75^As, ^111^Cd, ^137^Ba, ^208^Pb) and the stable isotopes of carbon (δ^13^C) and nitrogen (δ^15^N) obtained by del Rio-Lavín et al. (2022) [40] were used as input variables for the different models developed in this research. The methodology used to determine TEF and SIRA can be consulted in the original work of del Rio-Lavín et al. (2022) [40]. According to del Rio-Lavín et al. (2022) [40], the identification of the species of each sample was carried out using a SYBR™ Green post-PCR melting curve analysis [41].

After establishing the experimental data, each database (TEF—100 samples, SIRA—179 samples, and TEF + SIRA—64 samples) was divided into three different groups (Table 1).

Firstly, the database was divided into three random groups (shuffled sampling) to develop the different machine learning models. The first group corresponds to the training set (T); the data used here comprises 50% of the total dataset. This group aims to train multiple ML models to accurately predict the mussel’s geographical origin. The second group is composed of 25% of the total samples of the database used. This sample set is known as the validation group (V), which aims to select the best model within each ML model according to specific requirements. Finally, the last 25% was used as the query group (Q) to assess the performance of the selected best model and to evaluate power prediction on external data. In our research group, normally these groups are divided into 50%, 30%, and 20% (all this based on previous experience); however, in the present research, a different division has been adopted to make it more comparable to the data previously reported by del Rio-Lavín et al. (2022) [40].

Secondly, the alternative database division involves a 50%–25%–25% split but using stratified sampling; that is, creating a random subset of data while ensuring that the distribution of classes within each subgroup is approximately the same as in the overall dataset.

### 2.2. Machine Learning Approaches

In this research, to develop the random forest models, different hyperparameters were used to obtain the most accurate model possible: (i) number of trees and steps (from 1 to 200 with 199 steps in linear scale), (ii) maximum depth (from 1 to 200 with 199 steps in linear scale), (iii) pruning (true or false), (iv) pre-pruning (true or false), (v) selection criteria (gain ratio, Gini index, information gain, and accuracy), and (vi) voting strategy (confidence vote or majority vote).

The models developed in this study were approached from three different points of view: the first using the real variables of the input and output variables, while in the other two, the variables were normalised to reduce possible deviations of the data that could cause deficient results. The normalisation methods were range normalisation (from −1 to 1) and Z transformation (subscript Z). These models are identified by their R and Z subscripts, respectively. Both normalisations were first performed on the training samples and then applied to the validation and query samples. Therefore, in this research, three different configurations of random forest models were carried out for each database: RF, RF_R_, and RF_Z_.

Different options and hyperparameters were analysed to obtain the most accurate model possible. In this case, two SVM model types (C-SVC and nu-SVC—if possible) and two hyperparameters (C from approximately 9.77·10^−4^ and 1,048,576 in 30 linear or logarithmic—subscript L—steps and gamma from approximately 9.54·10^−7^ and 256 in 28 linear or logarithmic—subscript L—steps) were analysed. The SVM models were developed with real variable values and then with a normalisation process (range and Z transformation) (subscript Z). Therefore, in this research, six different support vector machine configurations were carried out for each database: SVM, SVM_L_, SVM_R_, SVM_R-L_, SVM_Z_, and SVM_Z-L_.

As stated above, ANN models consist of several nodes interconnected and distributed in different layers [42,43]. In the type of neural network developed in this research, the number of layers is three: the input layer, the hidden layer, and the output layer [37,43]. Within the input layer, the total number of neurons is dependent on the database used, so for the TEF database, the number of variables used is 14, for the SIRA database, it is 2; and for the TEF + SIRA database, it is 16. For models with reduced variables, the input variables depend on the selected database used, but the number is always 6. The hidden layer is composed of a total number of nodes that corresponds to the formula “*2n + 1*”, with “*n*” as the number of input variables.

Different hyperparameters were analysed: the number of training cycles (from 1 to 524,288 in 19 linear or logarithmic steps) and the decay (true or false). Therefore, in this research, six different support vector machine configurations were carried out for each database: ANN, ANN_L_, ANN_R_, ANN_R-L_, ANN_Z_, and ANN_Z-L_.

**Figure 1:**
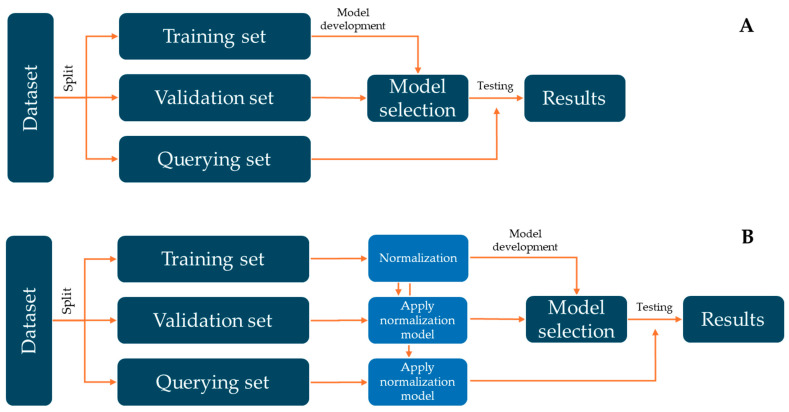
Flowchart of the process carried out to develop the different machine learning models. (**A**) shows the performance of ML models without the normalisation step. (**B**) represents the development of ML models with the normalisation step. Figure inspired by Rodríguez-Fernández et al. (2024) [44].

Finally, Figure 1 shows a general flowchart to develop machine learning models in this research.

### 2.3. Best Model Selection

In this study, accuracy and kappa were used. The accuracy value is reported as the ratio of correct predictions (where zero is the minimum value and one is the maximum value), and the kappa value is reported as a measure of the intervention of chance in predictions (where one is the desired value).

The best models within each approach (i.e., within the random forest group, the support vector machine group, or the artificial neural network group) will be chosen based on the highest accuracy value for the validation phase (this decision will also be based jointly on the kappa value for the same phase).

### 2.4. Equipment and Software

The computational equipment was an AMD Ryzen 9 7950X with 128 GB of RAM. The models presented here have been developed using Altair AI Studio Units 2025.1.0 (Altair Engineering Inc., Troy, MI, USA). Figures were created with Microsoft PowerPoint, and the plots were made with SigmaPlot 13.0 (Systat Software Inc., Palo Alto, CA, USA).

## 3. Results and Discussion

### 3.1. Models to Predict Region Using Shuffled Sampling

#### 3.1.1. TEF Models

These are the first prediction models developed, based on a random split maintaining the 50%–25%–25% distribution. In this case, the models were built using 14 input variables to predict the 8 different harvesting regions. As previously stated, the number of available samples with these 14 trace elements is 100, distributed evenly among all regions (10 samples each) except for Galicia and the Basque Country, where the number of samples collected increases to 20 (due to the presence of two locations in each of these regions). In this sense, the regions to be determined will be eight: Brittany (France), Algarve (Portugal), Galicia (Spain), the Basque Country (Spain), Catalonia (Spain), Emilia-Romagna (Italy), Bizerte (Tunisia), and Biobío (Chile). The distribution of these samples across the different phases is shown in Table 1.

For these developed models (Table 2, TEF models), four models were selected based on their accuracy values in the validation phase. These models include a random forest model (RF), which achieves an accuracy of 0.960 in the validation phase; a support vector machine model (SVM_R-L_) with a lower accuracy (0.880); and, finally, two artificial neural network models (ANN_Z_ and ANN_Z-L_) with accuracy values comparable to that of the random forest model. These accuracy values are accompanied by kappa values above 0.850 for the support vector machine model and 0.950 for the random forest model and the two artificial neural network models. These accuracies were extrapolated to the query phase, in which the models achieved slightly lower accuracy values: 0.880 for the random forest model, 0.800 for the two artificial neural network models, and a lower value of 0.720 for the support vector machine model. For all data, the accuracy values for these models vary between 0.900 (SVM_R-L_) and 0.960 (RF model), which gives an idea of the good general prediction power that the selected models have.

Although the overall accuracy values are high for all the data, it can be observed that, in the query phase, the SVM model and the ANN models show low accuracy values. Therefore, it can be said that only the random forest model would show a reasonably adequate performance when dealing with previously unseen samples.

The results obtained from the TEF models selected in this research suggest a general improvement in terms of accuracy compared with the models developed by del Rio-Lavín et al. (2022) [40]. The authors obtained, for their random forest model, an overall accuracy of 87.8% with a kappa value of 0.93, slightly lower than those achieved with the different models developed in this research, where accuracy values ranged from 90.0 to 96.0%, with kappa values between 0.884 and 0.953 (Table 2).

#### 3.1.2. SIRA Models

The next models developed used stable isotope ratios (δ^13^C and δ^15^N) as input variables. In this case, the number of available samples is 179, with Galicia and the Basque Country being the regions with the largest number of samples—40 each. As shown in Table 2 (SIRA models), five models were selected to present the highest values of that attitude in the validation phase (for each approximation). Once again, the random forest models, in this case in their three variants (RF, RF_R_, and RF_Z_), show the highest accuracy value (0.932), slightly lower than the RF-TEF model previously selected (0.960).

It can be observed that for both the support vector machine model and the selected ANN model, the validation accuracy falls to 0.841, the lowest value among all models developed so far. The good adjustments in the validation and training phases are not extrapolated to the query phase, where the accuracy values fall between 0.689 and 0.756. However, it can be noted that overall, that is, considering all data, the accuracy values remain relatively high (0.866), except for the artificial neural network model, which falls to 0.765.

When comparing the results of the SIRA models with those obtained from the TEF models, it becomes clear that the adjusted metrics decrease for the SIRA models across all phases. This is particularly evident in the query phase, where accuracy values fall from the 0.720–0.880 range to values between 0.689 and 0.756.

The loss of predictive power observed in the SIRA models could be explained by two factors. The first is the unequal distribution of the different regions across the model development phases. This becomes particularly evident with the heterogeneous distribution of samples, which increases for SIRA data (Table 1). Another possible explanation may be related to the uneven distribution of samples across different harvest seasons. As is well known, the harvest season has a significant influence on this property, and given that the data provided by the authors shows a certain imbalance, this could be responsible for the loss of predictive capability.

The results obtained from the SIRA models (accuracy values ranged from 76.5 to 86.6% with kappa values between 0.717 and 0.841—Table 2) suggest a general improvement in terms of accuracy compared with the models developed by del Rio-Lavín et al. (2022) [40] (overall accuracy of 80.5% with a kappa value of 0.81).

#### 3.1.3. TEF + SIRA Models

Due to the decline in predictive capacity observed in the models developed using stable isotope ratios as input variables, it was proposed to develop new models that combine these variables with the 14 input variables used in the trace element fingerprinting approach. It is therefore referring to the development of models with 16 input variables, which could, through this combination, improve the predicted performance of the selected models.

For these models, the total number of samples is 64, as shown in Table 1, and just as occurred with the other input variable distributions, the samples are imbalanced across the different phases of model construction.

Table 3 shows the models selected for each approach, considering the highest accuracy value obtained in the validation phase. As can be observed, the number of models that present a good performance increases to eight. Based on the results shown in the validation phase, it can be said that the combination of TEF and SIRA variables improves the model performance at this phase. The selected random forest models, representing the three different approaches (RF, RF_R_, and RF_Z_), achieve an accuracy value of 1. Next, the model with the second-highest accuracy value is the artificial neural network model (0.938), followed by four support vector machine models (SVM_R_, SVM_R-L_, SVM_Z_, SVM_Z-L_), each presenting accuracy values of 0.875.

Unfortunately, this high performance in the validation phase is not extrapolated to the query phase, given that the models with the highest accuracy values during the validation phase (1.000 and 0.938) are the ones that show the lowest accuracy values in the query phase (0.875); meanwhile, the SVM_R_, SVM_Z_, and SVM_Z-L_ models, which presented accuracy values of 0.875 in the validation phase, achieve accuracy values of 1 in the query phase.

The results obtained from the TEF + SIRA models selected in this research suggest a similar behaviour to that reported by del Rio-Lavín et al. (2022) [40] in their original research.

#### 3.1.4. TEF + SIRA Optimised Models

The relative improvement of the TEF + SIRA models in the validation and query phase may be due to the combined use of 16 input variables. However, it must be noted that some of these variables could introduce interferences that reduce the predictive power of the selected models, or they could increase the computational cost, as well as the costs associated with the sample analysis and the personnel costs. To avoid these, two approximation methods will be used:

- The first, using the variables proposed by del Rio-Lavín et al. (2022) [40], which chose four input TEF variables based on the Gini index (Pb, Ba, Mn, and Al) and the stable isotope ratios δ^13^C and δ^15^N. The choice of these trace elements detected in the shells is also supported by the Kruskal–Wallis test analysis carried out by del Rio-Lavín et al. (2022) (these four elements presented the most significant variations between the different harvesting sites [40]). With these six input variables, the TEF + SIRA reduced models were developed.
- The second way is based on the four TEF most important variables that were chosen according to their importance by the random forest models (TEF models) (the importance is given by the sum of the choice of a specific attribute supplied at a node [45]). In this case, the selected variables were Pb, B, Ba, and Mn, together with the stable isotope ratios δ^13^C and δ^15^N, were used to develop the models (TEF + SIRA reduced new models).

TEF + SIRA reduced models

The models developed with the 6 input variables (Pb, Ba, Mn, Al, δ^13^C, and δ^15^N) proposed by del Rio-Lavín et al. (2022) [40] are shown in Table 3 (TEF + SIRA reduced models). In this case, a total of 7 models were selected across the three approaches used: three random forest models (RF, RF_R_, and RF_Z_), two support vector machine models (SVM_Z_ and SVM_Z-L_), and two artificial neural network models (ANN_Z_ and ANN_Z-L_).

Among these models, the ones that showed the best performance in the validation phase are the three random forest models (accuracy of 1), followed by the two artificial neural network models (ANN_Z_ and ANN_Z-L_, 0.938), and finally the two selected support vector machine models (SVM_Z_ and SVM_Z-L_, 0.875). In the query phase, the random forest models achieve the best performance (0.938), together with the SVM_Z-L_. These random forest models also show very high accuracy values for all data (0.984).

Given the accuracy and kappa values for each phase, it can be said that the variable optimisation proposed by del Rio-Lavín et al. (2022) [40], that is, the use of Pb, Ba, Mn, Al, δ^13^C, and δ^15^N, has a positive impact on the predictive power of the models, not only when compared to the TEF + SIRA models but also when compared to models that use only the TEF or SIRA variables.

The results obtained from the best TEF + SIRA reduced models selected in this research (accuracy of 98.4% with a kappa value of 0.982—Table 3) suggest a slightly better performance than that reported by del Rio-Lavín et al. (2022) [40].

TEF + SIRA reduced new models

In this case, the models developed with the input variables (Pb, B, Ba, Mn, δ^13^C, and δ^15^N) are shown in Table 3 (TEF + SIRA reduced new models).

A total of 8 models were selected: the three random forest models (RF, RF_R_, and RF_Z_), four support vector machine models (SVM_R_, SVM_R-L_, SVM_Z_, and SVM_Z-L_), and one artificial neural network model (ANN_Z-L_) presented the best accuracy values in the validation phase.

The RF models present an accuracy value of 1 for the validation phase. These good accuracies are extrapolated to the query phase, where all models achieve a value of 0.875, a value slightly far from what would be ideal. The next best performance in the validation phase is obtained by the ANN_Z-L_ model, with an accuracy value of 0.938. This behaviour exhibited by the model in the validation phase, it is extrapolated to a high accuracy in the query phase, in this case again with an accuracy value of 0.938 with a kappa value of 0.922.

The optimisation of variables proposed in this research does not differ excessively, in terms of accuracy for the best models selected, from the proposed reduction by del Rio-Lavín et al. (2022) [40].

#### 3.1.5. General Assumptions About the Models Developed Using Shuffled Sampling

According to the results shown in Table 2 and Table 3, a series of statements can be made:

- First, among the models that use only one type of variable (TEF vs. SIRA models), the models that exhibit the highest accuracy are generally those developed with a larger number of input variables. It seems clear that a reduced number of variables (SIRA models) shows a certain inability to predict across the different categories. As already mentioned, this may be due both to the low number of input variables used and/or to issues associated with the unequal class distribution among the different data groups. One must also consider the influence that the collection of samples across the different seasons of the year may have.
- On the other hand (second), among the models that use both types of variables, the ones that present the best results are the TEF + SIRA reduced models (in other words, the models developed with the variables proposed by the authors). Considering variable reduction overall, it can be stated that the most important variable selection, aimed at reducing costs and computational load, generally produces better fits than the models that use all variables. This can be observed in the query phase results of the best selected models (selected based on the highest accuracy value in the validation phase, highlighted in grey in the tables). This behaviour could be attributed to variables that can inhibit the prediction power due to noise. Reducing the variables would eliminate this noise from the system.

### 3.2. Models to Predict Region Using Stratified Sampling

Based on the data presented in Table 1 and considering the variability due to random distribution into 50%, 25%, and 25%, the dataset was split in a way to ensure, as far as possible, a similar class distribution within each group. This approach aims to improve the performance achieved by the machine-learning models developed in Section 3.1.

#### 3.2.1. TEF Models

The first models developed are the models that use trace element fingerprints as input variables. Specifically, these models were developed using 14 input variables to predict any of the 8 harvest regions of the samples analysed.

In this case, as seen in Table 4 (TEF models), 7 models have been chosen as those that have the best accuracy value for the validation phase. These models are the three variations in the random forest models (RF, RF_R,_ and RF_Z_) that obtain an accuracy of 0.964, three support vector machine models (SVM_R-L_, SVM_Z_, and SVM_Z-L_) with accuracy values lower than the random forest models (0.821), and finally an artificial neural network model (ANN_Z-L_) with an intermediate accuracy (0.857) between the last two groups of models. These good accuracies are extrapolated to the query phase, in which the selected models obtain accuracy values higher than 0.810. Thus, in the case of the random forest models, they obtain accuracy values of 0.909, which are accompanied by kappa values higher than 0.891, which gives an idea of the good prediction power of these models for the query phase. On the other hand, two support vector machine models (SVM_Z_ and SVM_Z-L_) present the same accuracy values (0.909) as the random forest models, while the SVM_R-L_ model decreases its performance to 0.818, decreasing its kappa value to 0.785, adjustments that are identical to those provided by the ANN_Z-L_ model for the query phase. For all data, the accuracy values for these models vary between 0.910 (SVM_R-L_ and ANN_Z-L_) and 0.970 (RF models), which gives an idea of the good general prediction power that the selected models have.

#### 3.2.2. SIRA Models

The second models developed are the models that use the stable isotope ratio analysis as input variables. These models were developed using δ^13^C and δ^15^N as input variables to predict the harvest regions of the samples analysed. In this case, the number of available samples is 179, with Galicia and the Basque Country the regions with the most samples (40 each).

In this case, as can be seen in Table 4 (SIRA models), only 5 models have been selected because they present, within each approximation, the highest accuracy value for the validation phase. Once again, within RF models, the three variations (RF, RF_R_, and RF_Z_) obtain an accuracy of 0.911 (lower than the models developed using TEF data—0.964). Within the support vector machine models, on this occasion, only the model SVM_L_ was selected, presenting an accuracy value of 0.867, the same value obtained by the best ANNz model. For the query phase, a slightly different behaviour is observed concerning the validation phase. In this case, the model that has the best accuracy values is the SVM_L_ model with a value of 0.818, followed by the random forest models with a value of 0.795, and finally, the selected model that presents the worst accuracy value (0.750) for the query phase is the ANNz model. The kappa values for the selected models vary between 0.697 for the ANNz model and 0.786 for the SVM_L_ model.

Comparing the results obtained by the SIRA models with the selected models that use the TEF input variables, it can be seen that the use of stable isotope ratio data represents a loss of prediction power for the selected models, which, although for the validation phase they present a slight difference, it can be seen that for the query phase the fall in terms of accuracy is greater, a fact that can also be seen for all data in which it falls from a maximum value of 0.970 (for RF models TEF) to 0.883 for RFs and SVM_L_ model SIRA.

This loss in predictive power may be related to the uneven distribution of data across collection seasons. As is well known, the collection season has a strong influence on this property, and because the data provided by del Rio-Lavín et al. (2022) [40] already exhibit a certain imbalance, and because the distribution of samples across the different groups may also be unbalanced, this could therefore be partly responsible for this loss of predictive power.

#### 3.2.3. TEF + SIRA Models

Given the decline in the predictive power of models developed with stable isotope ratio input variables, it was proposed to develop models that use these variables together with the 14 trace element fingerprint input variables to study their behaviour and see if their joint use allows an improvement in the prediction of the models selected. In this case, the number of available samples is 64.

Table 5 shows the models selected (TEF + SIRA models) for each approximation, considering the best accuracy for the validation phase. 

As can be seen in this case, the number of models that present a better performance increases to seven, as occurred in the models developed with TEF variables. Based on Table 5, it seems clear that the use of both groups of variables allows an improvement in the accuracy value for the validation phase. This is visible in the adjustments obtained by the random forest and artificial neural network models, which achieve a correct classification of 1 (that is, 100%), while the previously developed models did not reach this value at any time. This group of selected models is made up of the three random forest models that achieve a correct classification value of 100%, two support vector machine models (SVM and SVM_L_) whose accuracy value reaches 0.944, and two artificial neural network models (ANN_Z_ and ANN_Z-L_) that also achieve 100% accuracy. Unfortunately, this good performance in the validation phase is not reflected in the query phase for the three random forests and the SVM and SVM_L_ models, since the results showed a decrease to an accuracy value of 0.846 and 0.692, respectively. In the case of artificial neural network models (ANN_Z_ and ANN_Z-L_), the accuracy increases up to 0.923, surpassing the accuracy of all the models developed so far (TEF and SIRA models).

For all data (TVQ), it can be observed that in general, the random forest (accuracy of 0.938) and support vector machine models (accuracy of 0.906) improve for this phase, the models developed only with the SIRA input variables (accuracy between 0.765 and 0.883) and worsen the accuracies presented by the models that use the TEF variables (accuracy between 0.910 and 0.970). However, the selected artificial neural network models (ANN_Z_ and ANN_Z-L_) improve the accuracy values obtained by the TEF and SIRA selected models, obtaining for all data an accuracy of 0.984 (higher than the accuracies obtained by the RF TEF models—0.970). In addition, these models (ANN_Z_ and ANN_Z-L_), which integrate TEF + SIRA variables, present for all data a kappa value of 0.982, which is also higher than the kappa values obtained by the random forest models using the TEF variables (0.965).

It seems clear that at least for the artificial neural network models, the use of the TEF + SIRA variables improves the performance of the selected models (it must be taken into account that the samples used in each of the groups of models selected—TEF, SIRA, and TEF + SIRA—are different, due to the nature of the data supplied, so this must be taken into account when making this statement).

#### 3.2.4. TEF + SIRA Optimised Models

TEF + SIRA reduced models

In this case, the models developed with the input variables (Pb, Ba, Mn, Al, δ^13^C, and δ^15^N) proposed by del Rio-Lavín et al. (2022) [40] are shown in Table 5 (TEF + SIRA reduced models).

A total of 10 models were selected from the three different approaches used. The three random forest models (RF, RF_R_, and RF_Z_), three support vector machine models (SVM_R-L_, SVM_Z_, and SVM_Z-L_), and four artificial neural network models (ANN_R_, ANN_R-L_, ANN_Z_, and ANN_Z-L_) have resulted in the best models in terms of accuracy value for the validation phase. All these models present an accuracy of 1 (that is, 100%) for the validation phase, which in the case of the SVM_R-L_ and SVM_Z_ models becomes 0.846 for the query phase, while for the rest of the models, the accuracy reaches a value of 0.923. For all data, the SVM models obtain the lowest accuracy values, with values of 0.938 and 0.969, while the rest of the models, that is, the models based on random forests and artificial neural networks, achieve an accuracy value of 0.984 (which is associated with kappa values of 0.982).

Given the accuracy and kappa values for each phase, it can be said that the variable optimisation proposed by del Rio-Lavín et al. (2022) [40], that is, the variables Pb, Ba, Mn, Al, δ^13^C, and δ^15^N, has a positive impact on the predictive power of the models (64 samples), not only when compared to the TEF + SIRA (64 samples) models but also when compared to models that use only the TEF (100 samples) or SIRA (179 samples) variables.

TEF + SIRA reduced new models

**Figure 2:**
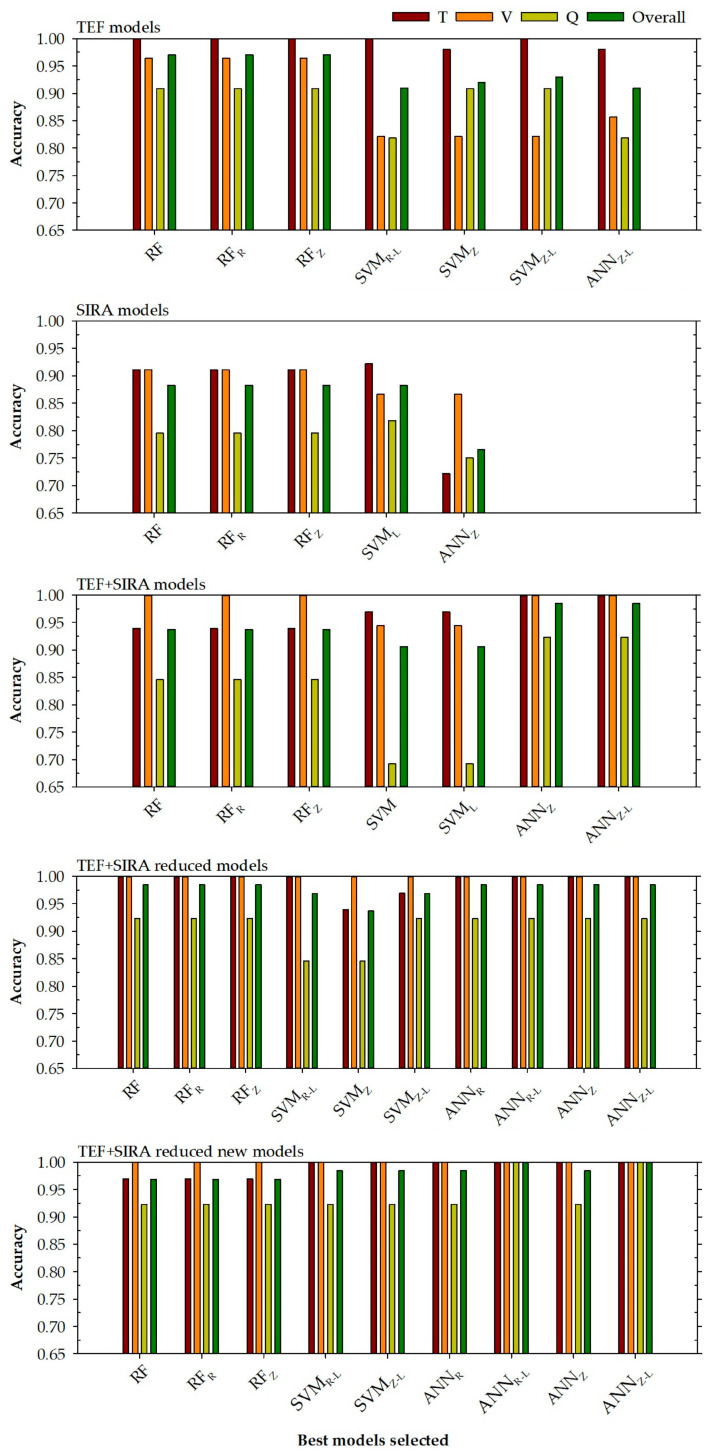
Accuracy levels achieved for each selected model to determine the region in training, validation, query phases, and all data (top right) based on the chosen input variables (top left). RF is a random forest model, SVM represents a support vector machine model, and ANN is an artificial neural network model. The normalisation methods were indicated by subscripts R (range normalisation from −1 to 1) and Z (Z transformation). Subscript L represents steps in a logarithmic scale.

In this case, the models developed with the input variables (Pb, Ba, Mn, Zn, δ^13^C, and δ^15^N) corresponding to the four TEF most important variables are shown in Table 5 and Figure 2 (TEF + SIRA reduced new models).

A total of 9 models were selected: the three random forest models (RF, RF_R_, and RF_Z_), two support vector machine models (SVM_R-L_ and SVM_Z-L_), and four artificial neural network models (ANN_R_, ANN_R-L_, ANN_Z_, and ANN_Z-L_) presented the best accuracy values for the validation phase. In the same way, as in the previous case, all the models present an accuracy value of 1 for the validation phase. These good accuracy adjustments are extrapolated to the query phase, in which all models except the ANN_R-L_ and ANN_Z-L_ models present an accuracy value of 0.923, while these two models present an accuracy of 100%. These two models, based on artificial neural networks, present an accuracy value of 1 for all data (with kappa values of 1). The rest of the models have accuracies of 0.969 or 0.984.

Given the accuracy and kappa values for each phase, it can be said that the variable optimisation proposed in this research provides slightly better results than the optimisation proposed by del Rio-Lavín et al. (2022) [40].

#### 3.2.5. General Assumptions About the Models Developed

Based on the values of the adjustment obtained (Table 4 and Table 5) and the graphs represented in Figure 2, for the different models developed, a series of statements can be made.

- In the first place, there is an important relationship with the different databases used. It can be seen from Figure 2 that the models that present better accuracy are generally those models that have more input variables. This can be seen by comparing the TEF with the SIRA models. Despite the number of different samples for both models (100 for TEF and 179 for SIRA), the reduced number of input variables used for the development of the SIRA models (δ^13^C and δ^15^N) does not allow for obtaining accuracy and kappa parameters of good quality, as those obtained by the TEF models. This is something that had been assumed could happen because the number of regions (8) may be too high for the small number of input variables that would be used to try to discriminate between them.
- Secondly, and this time comparing the models that use TEF and SIRA input variables to determine the different regions of origin of the mussel, it can be observed that those models that used a smaller number of input variables (4 TEF + 2 SIRA) present better results than the models that use all the variables (14 TEF + 2 SIRA). This gives the idea that among the TEF variables used, there are variables that inhibit the proper development of the models and only introduce noise into the system. Therefore, when a selection of the most representative TEF variables is made (TEF + SIRA reduced models or TEF + SIRA reduced new models), this noise is eliminated from the system, and the models with the optimised input variable selection produce better accuracy and kappa results.

### 3.3. Models to Predict Location Using Stratified Sampling

Given the good results obtained by the models developed to determine the harvest region, this research article proposes the development of new models that can determine the harvest location. In this sense, the locations to be determined will be ten: Bizerte Lagoon (Tunisia), Coliumo (Chile), Delta del Ebro, Mendexa, Mutriku, Ria de Arousa and Ria de Betanzos-Sada (Spain), Goro (Italy), Loquemeau (France), and Porto da Baleeira (Portugal).

#### 3.3.1. TEF Models

The first models developed used 14 input trace elements as input variables to predict the 10 harvest locations of the samples analysed. The number of samples was 100, distributed evenly among all locations (10 samples each).

In this case, Table 6 shows the eight selected TEF models chosen based on the accuracy value for the validation phase. These selected models are composed of the three random forest models (RF, RF_R_, and RF_Z_) with an accuracy value of 1.000, one support vector machine model (SVM_Z-L_) that presents an accuracy value of 0.867, and four artificial neural network models (ANN_R_, ANN_R-L_, ANN_Z_, and ANN_Z-L_) with an accuracy value of 0.933. These good accuracies shown by RF models are extrapolated to the query phase, which obtains an accuracy value of 0.950. The neural network models presented a decrease in prediction power for the query phase, obtaining results between 0.750 and 0.850. In this case, it seems clear that of the three different approaches, the models that offer the best results are the random forest models, not only because of their good adjustments for the validation (1.000) and query phase (0.950), but also for all data in which an accurate value of 0.990 is obtained (with a kappa value of 0.989).

#### 3.3.2. SIRA Models

The SIRA models were developed using δ^13^C and δ^15^N as input variables to predict the harvest location. The available samples were 179, distributed evenly among all regions (20 samples in each location), except for the locations of Bizerte Lagoon (12 samples), Goro (10 samples), and Loquemeau (17 samples).

It can be seen in Table 6 that the six best selected SIRA models presented the highest accuracy value for the validation phase. As can be seen, the accuracy level compared to the previous models drops significantly, in the same way as the models used to determine the region (Table 4). In this case, the model that presents the best accuracy for the validation phase is the SVM_L_ model with a value of 0.844, followed by the random forest models (0.800) and finally the ANN_R_ and ANN_R-L_ models with 0.778. These low adjustments even suffer a drop in the query phase, where the models that present the best performance are the ANN_R_ and ANN_R-L_ models (0.750), while the SVM_L_ model, which was the one that offered the best accuracy for the validation phase, now presents a value of 0.705. The RF models are the ones that present the worst accuracy values (0.614). Given the results, it was to be expected that the value for all data would also be low, with values between 0.799 (ANN_R_ and ANN_R-L_ models) and 0.866 (SVM_L_ model).

Comparing the results obtained in this approximation with the selected TEF models, it can be said that the use of stable isotope ratio analysis represents a high loss of prediction power for all phases analysed except the training phase. It is necessary to emphasise that in random forest models developed for the SIRA, there are certain samples whose confidence in the prediction is distributed equally among different categories. This means that for the same sample, depending on the situation, the model could predict one category or another. This is a serious drawback, which is why it is recommended that these random forest-based models not be used.

#### 3.3.3. TEF + SIRA Models

As in the case of the models to determine the region, and due to the decrease in predictive power of the models developed only with stable isotope ratios as input variables, it was proposed to develop models that use TEF and SIRA input variables. In this case, the number of samples was 64, with the locations of Goro and Bizerte Lagoon being those with the highest number of samples, 10. Table 7 shows the models selected (TEF + SIRA models) for each approximation, considering the best accuracy for the validation phase. 

As can be seen, the models that present a better attitude according to the accuracy value for the validation phase are nine: the three random forest models, the SVMR models, SVMR-L and SVMZ-L, and the artificial neural models, ANNR, ANNR-L, ANNZ, and ANNZ-L, showing these models an accuracy of 1.000. For the query phase of these selected models, the level of accuracy is very uneven, varying from 0.769 (SVMR-L, ANNR-L, and ANNZ models) to 0.923 for the RFZ model. The model that obtains the best level of prediction for all data is the RFZ model (0.984).

It seems clear that, at least for the random forest models, the use of the TEF + SIRA input variables improves the performance in most cases, the selected TEF and/or SIRA models (it must be considered that the samples used in each model (TEF—100, SIRA—179, and TEF + SIRA—64) are different).

#### 3.3.4. TEF + SIRA Reduced Models

Considering that the use of TEF + SIRA variables seems to improve the performance of the TEF and/or SIRA prediction models, the possibility of limiting the number of input variables is again studied to reduce the computational cost as well as associated costs.

The four most important TEF variables that were chosen according to their importance by the random forest models (TEF models). Therefore, in this new approach, the four most important TEF variables (Pb, Ba, Mn, and Al), together with the stable isotope ratios δ^13^C and δ^15^N, were used to develop the models (TEF + SIRA reduced new models). These variables are the same as those proposed by del Rio-Lavín et al. (2022) [40] for determining the harvest region.

Table 7 shows the models selected (TEF + SIRA optimised models) for each approximation. A total of eleven models were selected: the three random forest models (RF, RF_R_, and RF_Z_), four support vector machine models (SVM_R_, SVM_R-L_, SVM_Z_, and SVM_Z-L_), and four artificial neural network models (ANN_R_, ANN_R-L_, ANN_Z_, and ANN_Z-L_), according to the accuracy value for the validation phase. In this case, the RF and the ANN models present better accuracy (1.000) for the validation phase, while the SVM models present the lower value (0.938). For the query phase of these selected models, the level of accuracy varies from 0.769 (for the SVM models and the ANN_Z-L_ model) to 1.000 for the RF models. As expected, the models that obtain the best level of prediction for all data are the random forest models (1.000), followed by neural network models with an accuracy value between 0.938 and 0.984.

#### 3.3.5. General Assumptions About the Models Developed Using Stratified Sampling

**Figure 3:**
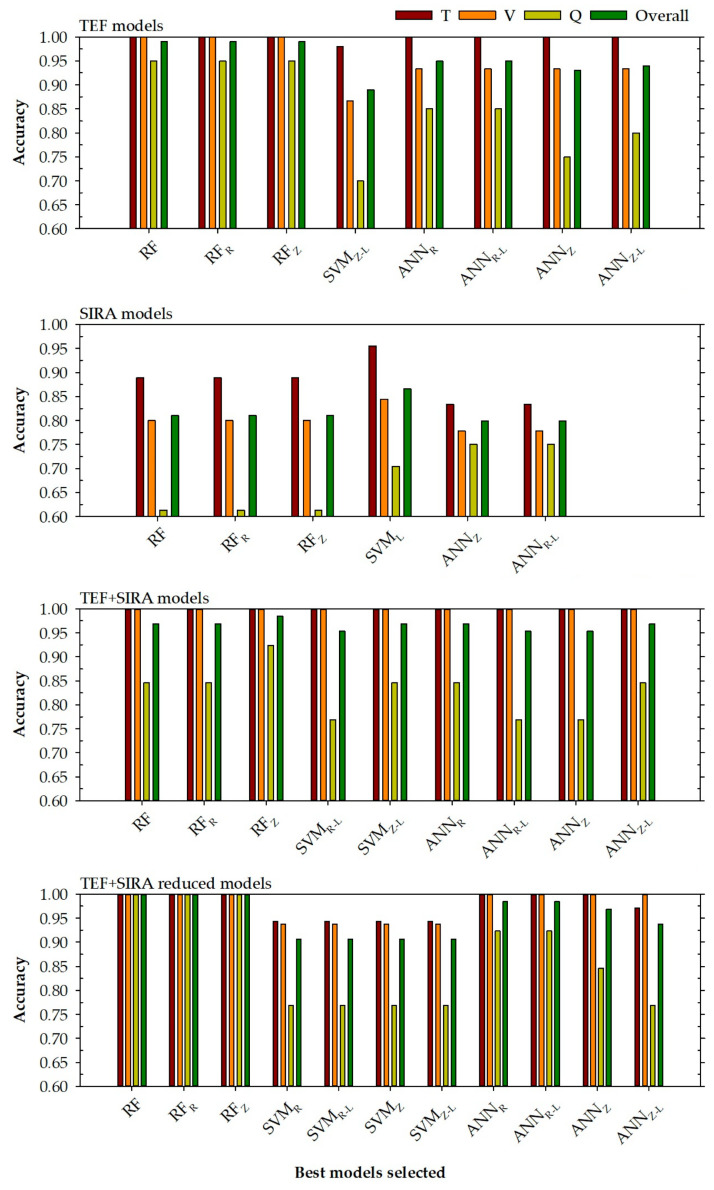
Accuracy levels achieved for each selected model to determine the location in training, validation, query phases, and all data (top right) based on the chosen input variables (top left). RF is a random forest model, SVM represents a support vector machine model, and ANN is an artificial neural network model. The normalisation methods were indicated by subscripts R (range normalisation from −1 to 1) and Z (Z transformation). Subscript L represents steps in a logarithmic scale.

According to the results shown in Table 6 and Table 7 and the graphs represented in Figure 3, a series of statements can be made:

- First, it can be seen from Figure 3 that the models that present the best accuracy are generally those models that have more input variables (this can be seen comparing the TEF with the SIRA models) or the models with the variable reduction (TEF + SIRA reduced models). Once again, it seems clear that the poor fits obtained in the SIRA models may be due to the low number of input variables and that they are not able to discern between the different locations. In addition to this, it is necessary to consider the problem that arose with the sample’s confidence being distributed equally among different categories.
- Secondly, showing the TEF + SIRA models and TEF + SIRA reduced models, it can be observed that the models with a small number of input variables present, in general, better results than the models that use all the variables (except for the SVM models, where a decline in prediction power is observed). This could be attributed to the existence of variables that can inhibit the prediction power of the models by introducing noise into the system. Reducing the variables would eliminate this noise from the system, thereby allowing the TEF + SIRA reduced models to improve their prediction power in all phases.
- Third, the performance of the models developed in this research is adequate, although there are models with low prediction power for the query phase. This happens especially for the SIRA models, in which the accuracy values for the query phase are in a very low range between 0.614 and 0.750.

### 3.4. Discussion

As previously stated, there are currently many procedures designed to identify the geographical origin. A widely used method, in this case, for identifying the origin of a product is the use of technology based on near-infrared spectroscopy (NIR). Thus, it could highlight the research carried out by Puleo et al. (2022) [46] to determine the origin of mussels (*Mytilus galloprovincialis*) from six different geographical areas located in Greece, Italy, Ireland, and Spain. Apart from the visual characteristics or NIR measurements, other characteristics, such as the mineral elements and/or stable isotopes, can be used to determine the geographical origin of food products such as mussels, oysters, or scallops, among others [1,19,47,48,49]. Therefore, it seems clear that the choice of these kinds of variables to determine the mussel’s geographical origin (*Mytilus galloprovincialis*) by del Rio-Lavín et al. (2022) [40] responds to the general feeling of the scientific community to determine the product’s geographical origin.

The results obtained by the different models developed using stratified sampling can be compared with the random forest models developed by del Rio-Lavín et al. (2022) [40]. In this sense:

- Seeing the models that use the TEF and SIRA variables (Figure 2), it can be seen that the models developed with the variables optimised based on the importance obtained by the input variables in the TEF models developed (TEF + SIRA reduced new models) generally present better adjustments for all phases compared to the variables proposed by del Rio-Lavín et al. (2022) [40]. This can be seen in the height of each bar for the selected models, where the models that use the selection of variables proposed in this research obtain higher values of accuracy for each phase. This is especially evident for the models ANN_R-L_ and ANN_Z-L_ that reach for all data an accuracy of 100%.
- The performance of the models developed in this research is adequate considering the adjustment provided by each selected model, although there are models in which a low prediction power is observed for the query phase. These are the cases of RF and ANN models of SIRA (79.5 and 75.0%, respectively) or the SVM models of TEF + SIRA (69.2%). In general, the models obtain accuracy values greater than 80.0% and, in many cases, greater than 90.0%.
- In addition to the good performance in the query phase, the models have demonstrated good accuracy adjustments for the general phase with very high values. In addition, it can be seen that the selected models present better adjustments overall than the models developed by del Rio-Lavín et al. (2022) [40]. For example, the TEF models developed by del Rio-Lavín et al. (2022) [40] present an overall accuracy of 87.8%, while the models selected in this research present for all data accuracy values between 91.0% (ANN_Z-L_) and 97.0% (RF models). In the case of the SIRA models, del Rio-Lavín et al. (2022) [40] obtained a general adjustment of 80.5%, while the models selected in this research present accuracies of 88.3%, except for the ANN_Z_ model, which drops to 76.5%.
- For the TEF + SIRA reduced model developed by del Rio-Lavín et al. (2022) [40], the authors report an overall accuracy of 97%. Using the same variables, the models selected in this research achieved values between 96.9% and 98.4% for all data (except for the SVM_Z_ model, whose accuracy only reaches 93.8%), which is a slight improvement over the models developed by del Rio-Lavín et al. (2022) [40]. Using the variable selection proposed in this research, the models obtain accuracy results between 96.9% and 100%, showing an improvement in the models, whose accuracy for all data reaches 100% in this case, the ANN_R-L_ and ANN_Z-L_ models.
- Summarising, it seems clear that the different machine learning approaches carried out in this research (especially the models based on random forests and artificial neural networks) improve the previous results of del Rio-Lavín et al. (2022) [40] and appear to be a suitable tool for determining the origin of the Mediterranean mussel (*Mytilus galloprovincialis*) in different locations around the world.

The performance of the models selected in this research can also be compared with other research found in the literature. In this sense, it can be researched to identify the region of origin of mussels, *Mytilus edulis*, using hydrogen, carbon, nitrogen, and oxygen compositions and stable isotope ratios [1]. Kang et al. (2022) [1] performed a principal component analysis and a linear discriminant analysis, which were fed with eight input variables (δ^2^H, δ^13^C, δ^15^N, δ^18^O, and H, C, N, and O) and observed that these signals were useful to determine the mussel’s origin (4 provinces) with an external validation accuracy rate of 92.3%. Saving the differences in the present research in terms of input variables (different combinations and optimisations) and the different regions studied, it can be seen that the models selected provide equal (92.3%) and better results (100%) (Table 5) than those reported by Kang et al. (2022) [1]. Some of the authors of the previously cited article [1] carried out similar research [47], but this time, using machine learning models, specifically through models of random forest or extreme gradient boosting algorithms, among others. According to Kang et al. (2023) [47], for the test data, the model that presented the best performance was the extreme gradient boosting model, reaching an accuracy of 93.75%. In this case, it can be observed that the use of machine learning models together with some modifications in the available data samples allowed for a slight increase in the accuracy of the model. The results presented by Kang et al. (2023) [47] (93.8%) are slightly superior to the best models selected in this research (92.3%) but inferior to the optimised new models (100%) proposed in this research (Table 5).

The results obtained in this research are also comparable and sometimes slightly superior to those shown in other research that has used oysters or scallops as a food product. An example is the research carried out by X. Zhang et al. (2019) [50] that used trace elements to determine the geographical origin of scallops (*Argopecten irradians, Chlamys farreri,* and *Patinopecten yessoensis).* X. Zhang et al. (2019) [50] used δ^13^C and δ^15^N values to identify seven locations in China using Fisher linear discriminant analysis (LDA). The LDA model presented an accuracy value of 92% for the testing samples. This result improves the models proposed in this research; however, it is necessary to take into account that the area studied by X. Zhang et al. (2019) [50] focused on the Bohai and Yellow Seas, while the data used in the present research are more heterogeneous, which could partly justify why the models presented in this article do not reach the level of accuracy of the models proposed by X. Zhang et al. (2019) [50]. On the other hand, Kang et al. (2024) [19] carried out research in which 18 elements were used to determine the geographical origin of Chinese oysters. Kang et al. (2024) used random forest and light gradient boosting machines, among other models, and obtained good overall accuracies with accuracy values that reached 96.8% [19]. Finally, Kang, Zhao, Peng et al. (2022) [48] used 14 mineral elements by different machine learning algorithms that included linear discriminant analysis, random forests, or support vector machines to determine the origin of scallops. In this case, linear discriminant analysis was able to obtain a 100% identification in the prediction phase, which showed that the use of elements such as fingerprints on scallops allows for determining the geographic origin of Chinese scallops [48]. The results achieved by these researchers present a perfect accuracy that fits with the best models developed in the present research.

In view of the results obtained in this research using shuffled sampling and stratified sampling, it can be stated that machine learning models based on random forests, support vector machines, and artificial neural networks are useful tools for determining the region and location of the Mediterranean mussel. However, the following points must also be considered:

- As evidenced by the results obtained by the different models developed in this research, it can be said that those models that only use the SIRA input variables present a low predictive power. This behaviour can be attributed to the variability in the stable isotope ratios due to the influence of the harvest seasons. Likewise, it is also necessary to indicate that the split procedure has a great influence on the results provided by the models. Thus, it has been observed that the models that have been developed under a shuffled sampling present worse results than the models developed under a stratified sampling.
- It would be very interesting, for future studies, to develop a database in which the samples were balanced in terms of region/location and sampling seasons. In addition, it would also be very interesting to investigate the influence that seasonality may have on the analytical results obtained.
- On the other hand, it would be interesting to have data from different years to evaluate the impact of the harvest year.
- It would be interesting to expand the number of input variables, especially for the SIRA models, to improve the accuracy of the selected models.
- In future research, it could be investigated how the use of other hyperparameter configurations (ranges, scales, or new hyperparameters can affect the result of the model) can affect the results obtained.
- Regarding training times and hardware resource consumption, the developed models were run on an AMD Ryzen 9 7950X with 128 GB of RAM. The computational cost associated with all training, validation, and query processes was almost entirely handled by the system’s processor. The execution time of the different models showed considerable variability, primarily due to the type of machine learning algorithm used. The lightest models were the support vector machine models, offering virtually instantaneous execution times. In contrast, the random forest models took the longest to execute, especially in scenarios with few input variables. Finally, with respect to artificial neural networks, execution times increased with a greater number of input variables. It would be very interesting, for a possible continuation of this study, to investigate how the use of a GPU might improve model execution times.

## 4. Conclusions

Determining the geographical origin of marine products, in this case Mediterranean mussels, is crucial for preventing fraud, ensuring food safety, and promoting sustainable management of diverse marine resources.

In this research, machine learning models based on random forest, support vector machine, and artificial neural network were used in combination with trace element fingerprinting (TEF) and stable isotope ratio analysis (SIRA) to determine the origin of *Mytilus galloprovincialis* (Mediterranean mussel) in eight regions and ten locations around the world. The trace elements were obtained from the shells, and the carbon and nitrogen isotopic ratios of the soft tissues of the mussels. Their use, individually, combined, or optimised, resulted in models with high predictive accuracy, especially highlighting artificial neural networks and random forests when they were developed with an optimised combination of the variables TEF and SIRA. The results obtained concluded that these machine learning approaches can be useful tools to determine the origin of Mediterranean mussels, having an important impact on food safety and sustainability.

Despite these good results, it is proposed that in future studies the accuracy of the models could be improved, especially those based on stable isotope ratios (the models with the worst results) by expanding the number of input variables, with the choice of different ranges and scales for the analysed hyperparameters, studying the influence of seasonality on the analytical results obtained or even evaluating the harvest year of the different mussels.

## Figures and Tables

**Table 1:** Distribution of each category across the training (T), validation (V), and query (Q) phases according to the type of division applied to the database.

Region (50%–25%–25%, shuffled sampling)													Region (50%–25%–25%, stratified sampling)										
	**TEF**				**SIRA**				**TEF + SIRA**				**TEF**				**SIRA**				**TEF + SIRA**		
	T	V	Q		T	V	Q		T	V	Q		T	V	Q		T	V	Q		T	V	Q
**Brittany**	5	1	4		8	4	5		6	1	1		5	3	2		9	4	4		4	2	2
**Algarve**	4	2	4		8	6	6		3	1	2		5	3	2		10	5	5		3	2	1
**Galicia**	9	7	4		21	12	7		3	1	6		10	5	5		20	10	10		5	3	2
**Basque Country**	13	3	4		19	12	9		4	5	1		10	5	5		20	10	10		5	3	2
**Catalonia**	5	1	4		11	4	5		1	2	2		5	3	2		10	5	5		3	1	1
**Emilia-Romagna**	4	3	3		5	1	4		6	2	2		5	3	2		5	3	2		5	3	2
**Bizerte**	7	2	1		9	2	1		8	1	1		5	3	2		6	3	3		5	3	2
**Biobío**	3	6	1		9	3	8		1	3	1		5	3	2		10	5	5		3	1	1
	50	25	25		90	44	45		32	16	16		50	28	22		90	45	44		33	18	13
	**Total**		100		**Total**		179		**Total**		64		**Total**		100		**Total**		179		**Total**		64
																							
**Location (50%–25%–25%, stratified sampling)**																							
	**TEF**				**SIRA**				**TEF + SIRA**														
	T	V	Q		T	V	Q		T	V	Q												
**Loquemeau**	5	3	2		9	4	4		4	2	2												
**Porto da Baleeira**	5	3	2		10	5	5		3	2	1												
**Ria de Arousa**	5	3	2		10	5	5		3	1	1												
**Ria de Betanzos**	5	3	2		10	5	5		3	1	1												
**Mendexa**	5	3	2		10	5	5		3	1	1												
**Mutriku**	5	3	2		10	5	5		3	1	1												
**Delta del Ebro**	5	3	2		10	5	5		3	1	1												
**Goro**	5	3	2		5	3	2		5	3	2												
**Bizerte lagoon**	5	3	2		6	3	3		5	3	2												
**Coliumo**	5	3	2		10	5	5		3	1	1												
	50	30	20		90	45	44		35	16	13												
	**Total**		100		**Total**		179		**Total**		64												

**Table 2:** Adjust parameters for the selected models to predict the region. The table shows accuracy (Acc.) and kappa values for the training (T), validation (V), training and validation (TV), query (Q), and all data (TVQ) phases according to the input variables used: TEF (trace element fingerprinting) and SIRA (stable isotope ratio analysis) models.

TEF Models										
	T		V		TV		Q		TVQ	
Model	Acc.	Kappa	Acc.	Kappa	Acc.	Kappa	Acc.	Kappa	Acc.	Kappa
RF	1.000	1.000	**0.960**	0.951	0.987	0.984	**0.880**	0.860	0.960	0.953
SVM_R-L_	1.000	1.000	0.880	0.855	0.960	0.953	0.720	0.672	0.900	0.884
ANN_Z_	1.000	1.000	**0.960**	0.952	0.987	0.984	0.800	0.766	0.940	0.930
ANN_Z-L_	1.000	1.000	**0.960**	0.952	0.987	0.984	0.800	0.766	0.940	0.930
**SIRA Models**										
RF	0.922	0.908	**0.932**	0.915	0.925	0.911	0.689	0.635	0.866	0.841
RF_R_	0.922	0.908	**0.932**	0.915	0.925	0.911	0.689	0.635	0.866	0.841
RF_Z_	0.922	0.908	**0.932**	0.915	0.925	0.911	0.689	0.635	0.866	0.841
SVM_Z-L_	0.878	0.856	0.841	0.808	0.866	0.841	**0.756**	0.713	0.838	0.809
ANN	0.767	0.720	0.841	0.797	0.791	0.745	0.689	0.630	0.765	0.717

**Table 3:** Adjust parameters for the selected models to predict the region. The table shows accuracy (Acc.) and kappa values for the training (T), validation (V), training and validation (TV), query (Q), and all data (TVQ) phases according to the input variables used: TEF + SIRA (trace element fingerprinting + stable isotope ratio analysis), TEF + SIRA reduced (input variables proposed by del Rio-Lavín et al. (2022) [40]), and TEF + SIRA reduced new models (input variables proposed in this research).

TEF + SIRA Models										
	T		V		TV		Q		TVQ	
Model	Acc.	Kappa	Acc.	Kappa	Acc.	Kappa	Acc.	Kappa	Acc.	Kappa
RF	1.000	1.000	**1.000**	1.000	1.000	1.000	0.875	0.847	0.969	0.964
RF_R_	1.000	1.000	**1.000**	1.000	1.000	1.000	0.875	0.847	0.969	0.964
RF_Z_	1.000	1.000	**1.000**	1.000	1.000	1.000	0.875	0.847	0.969	0.964
SVM_R_	1.000	1.000	0.875	0.848	0.958	0.951	**1.000**	1.000	0.969	0.964
SVM_R-L_	1.000	1.000	0.875	0.848	0.958	0.951	0.875	0.847	0.938	0.928
SVM_Z_	1.000	1.000	0.875	0.848	0.958	0.951	**1.000**	1.000	0.969	0.964
SVM_Z-L_	1.000	1.000	0.875	0.848	0.958	0.951	**1.000**	1.000	0.969	0.964
ANN_R-L_	1.000	1.000	0.938	0.925	0.979	0.976	0.875	0.847	0.953	0.946
**TEF + SIRA Reduced Models**										
RF	1.000	1.000	**1.000**	1.000	1.000	1.000	**0.938**	0.922	0.984	0.982
RF_R_	1.000	1.000	**1.000**	1.000	1.000	1.000	**0.938**	0.922	0.984	0.982
RF_Z_	1.000	1.000	**1.000**	1.000	1.000	1.000	**0.938**	0.922	0.984	0.982
SVM_Z_	0.969	0.962	0.875	0.845	0.938	0.926	0.875	0.845	0.922	0.909
SVM_Z-L_	0.969	0.962	0.875	0.845	0.938	0.926	**0.938**	0.922	0.938	0.927
ANN_Z_	1.000	1.000	0.938	0.923	0.979	0.976	0.813	0.768	0.938	0.928
ANN_Z-L_	1.000	1.000	0.938	0.923	0.979	0.976	0.813	0.768	0.938	0.928
**TEF + SIRA Reduced New Models**										
RF	1.000	1.000	**1.000**	1.000	1.000	1.000	0.875	0.847	0.969	0.964
RF_R_	1.000	1.000	**1.000**	1.000	1.000	1.000	0.875	0.847	0.969	0.964
RF_Z_	1.000	1.000	**1.000**	1.000	1.000	1.000	0.875	0.847	0.969	0.964
SVM_R_	0.969	0.962	0.875	0.845	0.938	0.926	0.875	0.845	0.922	0.909
SVM_R-L_	1.000	1.000	0.875	0.845	0.958	0.951	0.813	0.767	0.922	0.909
SVM_Z_	1.000	1.000	0.875	0.845	0.958	0.951	0.875	0.845	0.938	0.927
SVM_Z-L_	1.000	1.000	0.875	0.845	0.958	0.951	0.875	0.845	0.938	0.927
ANN_Z-L_	1.000	1.000	0.938	0.924	0.979	0.976	**0.938**	0.922	0.969	0.964

**Table 4:** Adjust parameters for the selected models to predict the region. The table shows accuracy (Acc.) and kappa values for the training (T), validation (V), training and validation (TV), query (Q), and all data (TVQ) phases according to the input variables used: TEF (trace element fingerprinting) and SIRA (stable isotope ratio analysis) models.

TEF Models										
	T		V		TV		Q		TVQ	
Model	Acc.	Kappa	Acc.	Kappa	Acc.	Kappa	Acc.	Kappa	Acc.	Kappa
RF	1.000	1.000	**0.964**	0.959	0.987	0.985	**0.909**	0.893	0.970	0.965
RF_R_	1.000	1.000	**0.964**	0.959	0.987	0.985	**0.909**	0.892	0.970	0.965
RF_Z_	1.000	1.000	**0.964**	0.959	0.987	0.985	**0.909**	0.893	0.970	0.965
SVM_R-L_	1.000	1.000	0.821	0.794	0.936	0.926	0.818	0.785	0.910	0.895
SVM_Z_	0.980	0.977	0.821	0.794	0.923	0.911	**0.909**	0.893	0.920	0.907
SVM_Z-L_	1.000	1.000	0.821	0.794	0.936	0.926	**0.909**	0.893	0.930	0.919
ANN_Z-L_	0.980	0.977	0.857	0.836	0.936	0.926	0.818	0.785	0.910	0.895
**SIRA Models**										
RF	0.911	0.895	**0.911**	0.894	0.911	0.895	0.795	0.757	0.883	0.861
RF_R_	0.911	0.895	**0.911**	0.894	0.911	0.895	0.795	0.757	0.883	0.861
RF_Z_	0.911	0.895	**0.911**	0.894	0.911	0.895	0.795	0.757	0.883	0.861
SVM_L_	0.922	0.908	0.867	0.841	0.904	0.886	**0.818**	0.786	0.883	0.861
ANN_Z_	0.722	0.664	0.867	0.840	0.770	0.723	0.750	0.697	0.765	0.716

**Table 5:** Adjust parameters for the selected models to predict the region. The table shows accuracy (Acc.) and kappa values for the training (T), validation (V), training and validation (TV), query (Q), and all data (TVQ) phases according to the input variables used: TEF + SIRA (trace element fingerprinting + stable isotope ratio analysis), TEF + SIRA reduced (input variables proposed by del Rio-Lavín et al. (2022) [40]), and TEF + SIRA reduced new models (input variables proposed in this research).

TEF + SIRA Models										
	T		V		TV		Q		TVQ	
Model	Acc.	Kappa	Acc.	Kappa	Acc.	Kappa	Acc.	Kappa	Acc.	Kappa
RF	0.939	0.930	**1.000**	1.000	0.961	0.955	0.846	0.822	0.938	0.928
RF_R_	0.939	0.930	**1.000**	1.000	0.961	0.955	0.846	0.822	0.938	0.928
RF_Z_	0.939	0.930	**1.000**	1.000	0.961	0.955	0.846	0.822	0.938	0.928
SVM	0.970	0.965	0.944	0.936	0.961	0.955	0.692	0.636	0.906	0.891
SVM_L_	0.970	0.965	0.944	0.936	0.961	0.955	0.692	0.636	0.906	0.891
ANN_Z_	1.000	1.000	**1.000**	1.000	1.000	1.000	**0.923**	0.910	0.984	0.982
ANN_Z-L_	1.000	1.000	**1.000**	1.000	1.000	1.000	**0.923**	0.910	0.984	0.982
**TEF + SIRA Reduced Models**										
RF	1.000	1.000	**1.000**	1.000	1.000	1.000	**0.923**	0.912	0.984	0.982
RF_R_	1.000	1.000	**1.000**	1.000	1.000	1.000	**0.923**	0.912	0.984	0.982
RF_Z_	1.000	1.000	**1.000**	1.000	1.000	1.000	**0.923**	0.912	0.984	0.982
SVM_R-L_	1.000	1.000	**1.000**	1.000	1.000	1.000	0.846	0.822	0.969	0.964
SVM_Z_	0.939	0.931	**1.000**	1.000	0.961	0.955	0.846	0.822	0.938	0.928
SVM_Z-L_	0.970	0.965	**1.000**	1.000	0.980	0.977	**0.923**	0.910	0.969	0.964
ANN_R_	1.000	1.000	**1.000**	1.000	1.000	1.000	**0.923**	0.910	0.984	0.982
ANN_R-L_	1.000	1.000	**1.000**	1.000	1.000	1.000	**0.923**	0.912	0.984	0.982
ANN_Z_	1.000	1.000	**1.000**	1.000	1.000	1.000	**0.923**	0.911	0.984	0.982
ANN_Z-L_	1.000	1.000	**1.000**	1.000	1.000	1.000	**0.923**	0.911	0.984	0.982
**TEF + SIRA Reduced New Models**										
RF	0.970	0.965	**1.000**	1.000	0.980	0.977	0.923	0.910	0.969	0.964
RF_R_	0.970	0.965	**1.000**	1.000	0.980	0.977	0.923	0.910	0.969	0.964
RF_Z_	0.970	0.965	**1.000**	1.000	0.980	0.977	0.923	0.910	0.969	0.964
SVM_R-L_	1.000	1.000	**1.000**	1.000	1.000	1.000	0.923	0.910	0.984	0.982
SVM_Z-L_	1.000	1.000	**1.000**	1.000	1.000	1.000	0.923	0.910	0.984	0.982
ANN_R_	1.000	1.000	**1.000**	1.000	1.000	1.000	0.923	0.910	0.984	0.982
ANN_R-L_	1.000	1.000	**1.000**	1.000	1.000	1.000	**1.000**	1.000	1.000	1.000
ANN_Z_	1.000	1.000	**1.000**	1.000	1.000	1.000	0.923	0.912	0.984	0.982
ANN_Z-L_	1.000	1.000	**1.000**	1.000	1.000	1.000	**1.000**	1.000	1.000	1.000

**Table 6:** Adjust parameters for the selected models to predict location. The table shows accuracy (Acc.) and kappa values for the training (T), validation (V), training and validation (TV), query (Q), and all data (TVQ) phases according to the input variables used: TEF (trace element fingerprinting) and SIRA (stable isotope ratio analysis) models. The symbol ^*^ represents those models that should be used with caution.

TEF Models										
	T		V		TV		Q		TVQ	
Model	Acc.	Kappa	Acc.	Kappa	Acc.	Kappa	Acc.	Kappa	Acc.	Kappa
RF	1.000	1.000	**1.000**	1.000	1.000	1.000	**0.950**	0.944	0.990	0.989
RF_R_	1.000	1.000	**1.000**	1.000	1.000	1.000	**0.950**	0.944	0.990	0.989
RF_Z_	1.000	1.000	**1.000**	1.000	1.000	1.000	**0.950**	0.944	0.990	0.989
SVM_Z-L_	0.980	0.978	0.867	0.852	0.938	0.931	0.700	0.667	0.890	0.878
ANN_R_	1.000	1.000	0.933	0.926	0.975	0.972	0.850	0.833	0.950	0.944
ANN_R-L_	1.000	1.000	0.933	0.926	0.975	0.972	0.850	0.833	0.950	0.944
ANN_Z_	1.000	1.000	0.933	0.926	0.975	0.972	0.750	0.722	0.930	0.922
ANN_Z-L_	1.000	1.000	0.933	0.926	0.975	0.972	0.800	0.778	0.940	0.933
**SIRA Models**										
RF ^*^	0.889	0.876	0.800	0.777	0.830	0.810	0.614	0.569	0.810	0.788
RF_R_ ^*^	0.889	0.876	0.800	0.777	0.830	0.810	0.614	0.569	0.810	0.788
RF_Z_ ^*^	0.889	0.876	0.800	0.777	0.830	0.810	0.614	0.569	0.810	0.788
SVM_L_	0.956	0.950	**0.844**	0.826	0.919	0.909	0.705	0.671	0.866	0.851
ANN_R_	0.833	0.814	0.778	0.752	0.815	0.794	**0.750**	0.722	0.799	0.776
ANN_R-L_	0.833	0.814	0.778	0.752	0.815	0.794	**0.750**	0.721	0.799	0.776

**Table 7:** Adjust parameters for the selected models to predict location. The table shows accuracy (Acc.) and kappa values for the training (T), validation (V), training and validation (TV), query (Q), and all data (TVQ) phases according to the input variables used: TEF + SIRA (trace element fingerprinting + stable isotope ratio analysis) and TEF + SIRA reduced models (input variables proposed in this research).

TEF + SIRA Models										
	T		V		TV		Q		TVQ	
Model	Acc.	Kappa	Acc.	Kappa	Acc.	Kappa	Acc.	Kappa	Acc.	Kappa
RF	1.000	1.000	**1.000**	1.000	1.000	1.000	0.846	0.827	0.969	0.965
RF_R_	1.000	1.000	**1.000**	1.000	1.000	1.000	0.846	0.827	0.969	0.965
RF_Z_	1.000	1.000	**1.000**	1.000	1.000	1.000	**0.923**	0.913	0.984	0.982
SVM_R-L_	1.000	1.000	**1.000**	1.000	1.000	1.000	0.769	0.738	0.953	0.947
SVM_Z-L_	1.000	1.000	**1.000**	1.000	1.000	1.000	0.846	0.824	0.969	0.965
ANN_R_	1.000	1.000	**1.000**	1.000	1.000	1.000	0.846	0.824	0.969	0.965
ANN_R-L_	1.000	1.000	**1.000**	1.000	1.000	1.000	0.769	0.738	0.953	0.947
ANN_Z_	1.000	1.000	**1.000**	1.000	1.000	1.000	0.769	0.738	0.953	0.947
ANN_Z-L_	1.000	1.000	**1.000**	1.000	1.000	1.000	0.846	0.824	0.969	0.965
**TEF + SIRA Reduced Models**										
RF	1.000	1.000	**1.000**	1.000	1.000	1.000	**1.000**	1.000	1.000	1.000
RF_R_	1.000	1.000	**1.000**	1.000	1.000	1.000	**1.000**	1.000	1.000	1.000
RF_Z_	1.000	1.000	**1.000**	1.000	1.000	1.000	**1.000**	1.000	1.000	1.000
SVM_R_	0.943	0.936	0.938	0.929	0.941	0.934	0.769	0.738	0.906	0.895
SVM_R-L_	0.943	0.936	0.938	0.929	0.941	0.934	0.769	0.738	0.906	0.895
SVM_Z_	0.943	0.936	0.938	0.929	0.941	0.934	0.769	0.738	0.906	0.895
SVM_Z-L_	0.943	0.936	0.938	0.929	0.941	0.934	0.769	0.738	0.906	0.895
ANN_R_	1.000	1.000	**1.000**	1.000	1.000	1.000	0.923	0.913	0.984	0.982
ANN_R-L_	1.000	1.000	**1.000**	1.000	1.000	1.000	0.923	0.913	0.984	0.982
ANN_Z_	1.000	1.000	**1.000**	1.000	1.000	1.000	0.846	0.827	0.969	0.965
ANN_Z-L_	0.971	0.968	**1.000**	1.000	0.980	0.978	0.769	0.740	0.938	0.930

## Data Availability

The raw data supporting the conclusions of this article will be made available by the authors on request.

## Abbreviations

The following abbreviations are used in this manuscript:

ANN	Artificial neural network using a linear scale
ANN_L_	Artificial neural network using a logarithmic scale
ANN_R_	Artificial neural network using range normalisation
ANN_R-L_	Artificial neural network using range normalisation and a linear scale
ANN_Z_	Artificial neural network using Z transformation
ANN_Z-L_	Artificial neural network using Z transformation and a linear scale
CARTS	Classification and regression trees
LDA	Linear discriminant analysis
ML	Machine learning
Q	Query group
RF	Random forest using a linear scale
RF_R_	Random forest using range normalisation
RF_Z_	Random forest using Z transformation
SIRA	Stable isotope ratio analysis
SVM	Support vector machine using a linear scale
SVM_L_	Support vector machine using a logarithmic scale
SVM_R_	Support vector machine using range normalisation
SVM_R-L_	Support vector machine using range normalisation and linear scale
SVM_Z_	Support vector machine using Z transformation
SVM_Z-L_	Support vector machine using Z transformation and linear scale
T	Training group
TEF	Trace element fingerprinting
V	Validation group
